# Knowledge, Attitudes, and Practices on Infection Control Measures in Stomatology Students in Lima, Peru

**DOI:** 10.1155/2018/8027130

**Published:** 2018-09-12

**Authors:** Oscar Silva, Silvia Palomino, Ada Robles, Jorge Ríos, Frank Mayta-Tovalino

**Affiliations:** ^1^Department of Stomatology School, Universidad Privada San Juan Bautista, Lima, Peru; ^2^Professor of the Stomatology School, Universidad Privada San Juan Bautista, Lima, Peru; ^3^Academic Coordinator of the Stomatology School, Universidad Privada San Juan Bautista, Lima, Peru; ^4^Academic Coordinator of the Master of Public Health, Universidad Privada San Juan Bautista, Lima, Peru

## Abstract

**Introduction:**

The level of knowledge, attitudes, and practices with respect to infection control measures in undergraduate stomatology students is not well understood; that is why these variables were evaluated in the students of the Universidad Privada San Juan Bautista between September and November of 2017.

**Methods:**

A cross-sectional study was carried out. A total of 347 students from the Ica, Lima Norte, and Chorrillos campuses were evaluated. The sample was calculated using the statistical formula of comparison of means. The questionnaire of the CDC (Center of Contagious Diseases) was used to measure the variables described.

**Results:**

It was observed that 72.05% of the students preferred to use oral rinsing before starting a treatment, 72.62% used the autoclave as the primary equipment to sterilize their instruments in the dental clinic, 95.10% considered that isolation is important in the control of the infection, 46.40% affirmed that tuberculosis is the most infectious disease, and only 26.51% considered it to be hepatitis B. On the other hand, it was found that the scores of knowledge, attitudes, and practices were 2.74 ± 2.16, 3.59 ± 0.88, and 3.59 ± 1.73, respectively.

**Conclusions:**

The level of knowledge was low among the students evaluated; however, as far as the level of practices and attitudes was high, even so, this topic must be reinforced so that stomatology students reflect on the importance of the risks that exist to get infected with any disease inside the dental office, as outside of it.

## 1. Introduction

The dental staff in these times are exposed to various risk factors that can lead to the spread of countless diseases that are transmissible through various types of fluid such as saliva and blood, especially hepatitis B and HIV that are considered major public health problems [1]. There are several factors that will directly impact the level of knowledge, attitudes, and practices of dental personnel to care for the various patients with infectious contagious diseases; therefore, it is essential that students handle good theoretical and practical concepts that improve the quality of care and reduce the prevalence of cross infections in the dental practice [2]. This risk can be increased by accidental injuries during dental treatment; therefore, a safety precaution work philosophy and the practice of infection control among these students should be implemented. This culture is the responsibility of the dental schools because they must guarantee the adequate measures of infection control and, above all, how to handle these situations of risk [1, 2].

The measures for the control of infectious diseases are an aspect that must be taken very seriously in dentistry, because in the environment where the dentist works, in sharp instruments, and in invasive procedures, various pathogens are present [3]. Therefore, it is essential to provide better information on protection and biosecurity and to motivate everyone to continue complying with the infection control standards in dentistry [4]. The expositions of the dentists by these risk factors are important to identify them for the creation of some sanitary strategy that prevents the control of infections in them [5].

Many of the injuries in dental practice are related to biohazard agents because they are doors of entry to serious and deadly diseases which will come into contact with a patient, and the most important and most prevalent diseases are HIV, hepatitis B, hepatitis C, and tuberculosis. According to evidence, dental students are the ones who are more prone to contract any disease due to contact with blood and other fluids [6]. In the area of dentistry, when performing various procedures, students do not take some basic principles of biosafety and knowledge of infection management; in addition, the field of work to perform a dental procedure is narrow and has poor visibility [7].

In dental practice, there are many students who have the skill in handling some sharp instruments and others who are more vulnerable to suffer some cut and get some disease [8]. For instance, according to a recent study, the places where most injuries occur are the fingers of the hand, followed by arms, palm, thigh, leg, foot, and cheeks [9]. These injuries occur when having a small space, when making sudden movements on the part of the patient or of the own practitioner, and when using sharp and cutting materials [10]. The knowledge about the etiological agents has evolved in such a way that the treatments and related factors have allowed to establish measures to reduce the health risks [11].

Therefore, the purpose of this research was to evaluate the level of knowledge, attitudes, and practices regarding the infection control measures in undergraduate stomatology students of the Universidad Privada San Juan Bautista, Lima, Peru, during the year 2017.

## 2. Materials and Methods

This study was approved under the permission of the professional ethics and bioethics committee of the San Juan Bautista Private University with code no. CEPB-FCS 0016. It also followed the guidelines for epidemiological, descriptive studies according to the STROBE guidelines (Strengthening The Reporting of Observational Studies in Epidemiology). All the research was carried out according to the principles expressed in the Declaration of Helsinki. Finally, all the participants signed their informed consent.

A total of 347 undergraduate stomatology students were evaluated, which were evaluated in all the campuses (Ica, Lima Norte, and Chorrillos) september november 2017. The sample size was calculated based on the scores obtained in the pilot test of the level of knowledge, attitudes, and practices using the mean comparison formula by software Stata 12.0, establishing a level of significance (alpha) of 0.05 and a power of test (beta) of 0.80.

The participants were selected in strict compliance with the following criteria:

  Inclusion criteria:
  (i) Undergraduate students of the Stomatology School of the UPSJB
  (ii) Students who agree to sign the informed consent
  (iii) Students in good physical and mental condition
  Exclusion criteria:
  (i) Students who do not want to participate in the study
  (ii) Students with a diagnosis of an infectious contagious disease

For the application of the instrument, the value of 0 was assigned to an incorrect answer and the value of 1 was assigned to a correct answer for each item. It was also obtained that the maximum score for knowledge was 6, for attitudes 4, and practices 5, according to the questions that were made within the questionnaire. It is worth mentioning that this methodology was repeated according to Singh et al. [12].

For the evaluation of quantitative variables such as the level of knowledge, attitudes, and practices, the mean and the standard deviation were obtained. On the other hand, for the descriptive analysis, we used the percentage (frequencies) of the qualitative variables such as sex, academic cycle, and university headquarters; likewise, the Pearson chi-square test was used for the bivariate statistical analysis using the Stata 12.0 software, establishing a probabilistic value of *p* < 0.05.

## 3. Results

When evaluating the sociodemographic characteristics of the students of the UPSJB during 2017, the cycle variable related to the sex variable was obtained as a result that the sixth cycle of the female reached the highest number of respondents with 15.3% (*n*=35) unlike the third cycle of the male sex that obtained 13.4% (*n*=16). Regarding the headquarters, a variable related to the sex, it was evidenced that in the Lima Norte and Chorrillos sites, the female sex was the most prevalent with 36.8% (*n*=84) with respect to the headquarters of Chorrillos where the male sex only obtained 31.0% (*n*=45), and the results are shown in Table 1.

When evaluating the association between knowledge, attitudes, and practices regarding infection control measures in undergraduate stomatology students of the UPSJB during 2017 according to sex, no statistical association was found with the level of significance (*p* > 0.05) between the questions (P1–P15) shown in Table 2.

However, when determining this relationship according to cycle, it was found that there is statistical association in question P3 (prefer to use oral rinse before starting any procedure of a treatment) with *p*=0.004, question P6 (which of the following options are used to sterilize the instruments in the dental clinic) with *p* < 0.001, question P7 (minimum time required for autoclaving) with *p*=0.001, question P8 (temperature for autoclaving) with *p*=0.038, question P9 (which of the following has the highest rate of transmission through of saliva) with *p* < 0.001, question P10 (what immediate action should be taken in case of direct blood contact with a patient with HIV) with *p*=0.019, question P12 (as a dentist, what protective measures taken to prevent an injury) with *p*=0.009, question P14 (ineffective sterilization during clinical practice can transmit the infection from one patient to another) with *p*=0.001, and finally question P15 (apart from sterilization of the instrument, disinfection of the dental chair of the dental office clinic is required) with *p*=0.037 (Table 3).

For the score, the value of 1 was placed for each correct answer and 0 for an incorrect one, following the methodology used in the study of Liege et al. [10]. The mean and the standard deviation were evaluated on knowledge, attitudes, and practices regarding the infection control in undergraduate stomatology students of the UPSJB where the values of 2.74 (2.16), 3.59 (0.88), and 3.59 (1.73), respectively, were found, thus noting that the score of the level of knowledge was the lowest, compared to the level of attitudes and practices that were higher with respect to the preclinical, clinical, and internship cycles (Table 4).

## 4. Discussion

The Peruvian dental university system is currently focusing on developing competencies related to biosecurity issues given that there is currently a high prevalence of contagious infectious diseases in Peru; this added to the large number of immigrants who are residing in the country. Therefore, the Peruvian students of stomatology must be aware and prepared to attend all kinds of patients at risk. There is a clear difference between the level of knowledge of the novice students of preclinical, clinical, and hospital internship courses; this is obviously due to the academic maturity that the student is experiencing throughout his academic life. For this reason, it is essential to create awareness among students of the first cycles of studies on the prevention and control of infections in stomatology [12].

The most amazing result of the study was that, in this research, the level of knowledge, attitudes, and practices regarding infection control measures in undergraduate dentistry students were evaluated because the percentage of being infected with any disease in dentistry is higher compared to other areas of the sciences. There is a very narrow field of work, also due to the contact of various sharp materials and the constant movement of the patient; that is why in studies performed to dentists, it was evidenced that the disease to be contagious more easily is the hepatitis B virus with 9%, comparing it with the diseases caused by the hepatitis C virus with 1.4%, followed by the human immunodeficiency virus varying from 0 to 0.8% [4].

The level of knowledge and attitudes about the measures of prevention and control of stomatology infection was relatively poor among the students evaluated. The reasons could be due to the fact that during its learning stage, there is inadequate feedback on biosecurity issues. When analyzing the results obtained, it was determined that it differs from other studies since in the present investigation it was found that the level of knowledge obtained the lowest score and the level of attitude and practice, on the contrary, reached the highest scores. However, in the study by Singh et al. [12] on knowledge, attitudes, and practice with respect to infection control measures among dental students in central India, they found that the attitude reached the highest score and the level of knowledge and practice was poor. On the other hand, Askarian and Assadian [13] in a study on infection control practice among dental professionals at a dental school in Shiraz, Iran, found that the level of practice was the lowest, compared to the level of attitude that was highest, and knowledge reached an average score. These differences occur because there is a low or inadequate training on infection control, biosecurity, and cross-disease, in addition to a lack of focus on this issue.

Therefore, to reduce or prevent the transmission of certain viruses and bacteria in the oral health staff, strict adherence to the biosafety regulations for infection control must be applied [12, 13].

In the oral health staff, strict adherence to the biosafety regulations for infection control must be applied [12, 13]. In the study of the evaluation of the level of knowledge, attitudes, and practices regarding measures of infection control in students according to sex, it was evidenced that the highest prevalence was in the female sex of the sixth cycle with 15.3% (*n*=35); this resembles the research carried out by some studies [3, 14] in which of 135 students, 60% represented the female sex in a study on knowledge, perception of risk, and attitudes of the students of dentistry with respect to HIV/AIDS. Other similar studies with respect to the sex variable are given in a study by Sadeghi and Hakimi [15] on knowledge and attitudes of Iranian dentistry students towards patients with HIV/AIDS, where of 455 usable surveys, 65.4% represented female sex since the vast majority of dentistry students in Iran are women. Another similar study is that of Abdullah [16] about a survey of needles and other acute injuries among 230 undergraduate dental students, 72.1% (*n*=166) were female, and 27.9% (*n*=64) were male. However, in the study conducted by Askarian and Assadian [13] on infection control practices among dental professionals in Shiraz Dentistry School, Iran, it is shown that of the 152 respondents, 78 (51.3%) were male, which shows that compared to the other studies mentioned above, the male gender was higher, 37 were members of the faculty (24 that attended and 13 residents), and 25 students were of the sixth cycle, 21 students were of the fifth cycle, and 69 students belonged to the fourth cycle of education, respectively; however, women (5.5) obtained the highest score in terms of practice compared to men (4.5) (*p* < 0.05).

When assessing the level of knowledge, attitudes, and practices regarding infection control measures in students regarding the cycle, it was subdivided into 3 groups (preclinical, clinical, and internship). It resembles the study conducted by Aggarwal and Panat [17] on knowledge, attitude, and behavior in the management of patients with HIV/AIDS in a group of Indian dentistry students; the group of 367 students was divided into freshmen with 80%, second year with 84%, third year with 72%, fourth year with 88%, and students who do internships with 75%. Finally, in another study conducted by Lopes et al [18] on hepatitis B, knowledge, vaccination status, and seroconversion on the part of the dental students of a public university were evaluated in 179 students who were enrolled in the 3rd cycle up to the 9th cycle, determining a high prevalence. For example, according to some studies, many patients have some fear of treating HIV patients, even taking biosecurity measures and specific conditions. Although this is not ideal from an ethical point of view, for a discriminatory topic, knowledge and attitudes were positively correlated [19, 20]. Although this is not ideal from an ethical point of view, for a discriminatory topic, knowledge and attitudes correlated in a positive way [19, 20].

The level of knowledge, attitudes, and practices on infection control measures was poor among undergraduate students, so it is suggested to conduct a rigorous training given that these results should put on alert the university professors of the stomatology schools of the country; these findings are similar to those described by other authors [12, 21, 22].

Within the limitations of this research was that little evidence was found with similarities in the results, because there is poor information on this subject, in addition that the level of knowledge of the students was low, due to the lack of reinforcement of the subject while the students advanced in the academic curriculum. Another limitation of this study was that the variation of self-reported attitudes towards the management of infectious diseases was relatively variable. This indicates that the level of knowledge, practices, and attitudes of stomatology students is affected by many factors difficult to control. However, the clearest advantage of this study was that the results reflect that Peruvian students have some experience in the control and management of this type of patients; therefore, the methodological design should be improved by conducting follow-up research to corroborate the experience of these students in time. It should be mentioned that multivariate statistical analyses should be carried out to help adjust and control confounding covariates.

The importance of this study was to evaluate the application of infection control measures within the dental clinic or any other dental office and what is the probability of infection in stomatology students by not keeping in mind the basic biosecurity principles. This study also has epidemiological impact because there are infectious diseases that occur within the office through a method of cross infection between dentistry students and patients who come to the consultation, not knowing if they suffer or are carriers of any disease.

## 5. Conclusions

In conclusion, this research found a worrying lack of knowledge and attitudes related to the control and prevention of infectious contagious diseases, both in preclinical, clinical, and hospital boarding students. Therefore, emphasis should be placed on student learning about the transmission of infections in the health sciences and, above all, on the importance of teaching this topic correctly in the first cycles so as not to forget the basic knowledge of biosafety and management of infection control in the future.

## Figures and Tables

**Table 1:** Sociodemographic characteristics of undergraduate stomatology students by sex, cycle, and headquarters.

Groups		Sex	
		Male, *n* (%)	Female, *n* (%)
Semester	I	7 (5.8)	16 (7.0)
	II	15 (12.6)	31 (13.6)
	III	16 (13.4)	25 (10.9)
	IV	15 (12.6)	24 (10.5)
	V	13 (10.9)	20 (8.7)
	VI	15 (12.6)	35 (15.3)
	VII	11 (9.2)	17 (7.4)
	VIII	7 (5.8)	22 (9.6)
	IX	9 (7.5)	12 (5.2)
	X	11 (9.2)	26 (11.4)

Headquarters	Ica	37 (31.0)	60 (26.3)
	Lima Norte	37 (31.0)	84 (36.8)
	Chorrillos	45 (31.0)	84 (36.8)

**Table 2:** Association between knowledge, attitudes, and practices regarding infection control measures in undergraduate stomatology students according to gender.

Question	Answer	Gender Male, *n* (%)	Female, *n* (%)	*p* ^*∗*^
Q1: Do you wash your hands before and after patient examination?	Yes	116 (97.4)	220 (96.4)	0.737
	No	3 (2.5)	8 (3.51)	
Q2: With what do you wash your hands?	Plain soap	83 (69.7)	161 (70.6)	0.984
	Detergent	1 (0.8)	2 (0.8)	
	Antiseptic solution	35 (29.4)	65 (28.5)	
Q3: Do you prefer oral mouth rinse before commencement of any treatment procedure?	Yes	90 (75.6)	160 (70.1)	0.282
	No	29 (24.3)	68 (29.8)	
Q4: Do you think isolation is important in infection?	Yes	112 (94.1)	218 (95.6)	0.540
	No	7 (5.8)	10 (4.3)	
Q5: With which of the following vaccines have you been vaccinated?	Hepatitis B	26 (21.8)	37 (16.2)	0.590
	Tetanus	39 (32.7)	85 (37.2)	
	Tuberculosis	52 (43.7)	103 (45.1)	
	None	2 (1.6)	3 (1.3)	
Q6: Which of the following do you use to sterilize the instruments? In dental clinic?	Autoclave	83 (69.7)	169 (74.1)	0.686
	Boiling	30 (25.2)	49 (21.4)	
	Washing	6 (5.0)	10 (4.3)	
Q7: Minimum time required for sterilization in autoclave?	5 min	2 (1.6)	9 (3.9)	0.340
	10 min	32 (26.8)	50 (21.9)	
	15 min	85 (71.4)	169 (74.1)	
Q8: Temperature for sterilization in autoclave?	100°C	7 (5.8)	16 (7.0)	0.833
	120°C	52 (43.7)	104 (45.6)	
	150°C	60 (50.4)	108 (47.3)	
Q9: Which of the following has the highest rate of transmission via saliva?	Hepatitis B	26 (21.8)	59 (25.8)	0.744
	AIDS	23 (19.3)	41 (17.9)	
	Tuberculosis	55 (46.2)	106 (46.4)	
	Do not know	15 (12.6)	22 (9.6)	
Q10: What immediate action should be taken in case of direct blood contact with an HIV patient?	Anti-HIV immunoglobulins	28 (23.5)	64 (28.0)	0.096
	Anti-HIV drugs	9 (7.5)	33 (14.4)	
	Blood tests	62 (52.1)	91 (39.9)	
	Do not know	20 (16.8)	40 (17.5)	
Q11: Odds of HIV transmission after a single contaminated needlestick injury?	0.1%–0.4%	10 (8.4)	16 (7.0)	0.437
	1%–4%	18 (15.1)	22 (9.6)	
	10%–40%	25 (21.0)	54 (23.6)	
	70%–90%	66 (55.4)	136 (59.6)	
Q12: As a clinician, what protective measures do you take to prevent yourself from injury?	Face mask and gloves	10 (8.4)	8 (3.5)	0.13
	Eyewear	18 (15.1)	38 (16.6)	
	Protective clothing	4 (3.3)	3 (1.3)	
	All the above	87 (73.1)	179 (78.5)	
Q13: After use of gloves for a patient, what do you do with them?	Dispose them	119 (100)	226 (99.1)	0.306
	Reuse them after wash	0 (0)	2 (0.8)	
	Reuse them after sterilization	0 (0)	0 (0)	
Q14: Ineffective sterilization during clinical practice can transmit the infection from one patient to another?	Yes	105 (88.2)	208 (91.2)	0.375
	No	1 (0.8)	4 (1.7)	
	Do not know	13 (10.9)	16 (7.0)	
Q15: Apart from instrument sterilization, disinfection of dental chair, clinic, dental office is required	Yes	107 (89.9)	211 (92.5)	0.586
	No	4 (3.3)	4 (1.7)	
	Do not know	8 (6.7) 105 (88.2)	13 (5.7. 208 (91.2)	

^*∗*^Pearson's chi-square test. Level of significance: *p* < 0.05.

**Table 3:** Association between knowledge, attitudes, and practices regarding infection control measures in undergraduate stomatology students according to cycle.

Question	Answer	Cycle			*p* ^*∗*^
		Preclinical, *n* (%)	Clinical, *n* (%)	Internship, *n* (%)	
Q1: Do you wash your hands before and after patient examination?	Yes	143 (41.2)	136 (39.1)	57 (16.4)	0.337
	No	6 (1.7)	4 (1.5)	1 (0.2)	
Q2: With what do you wash your hands?	Plain soap	104 (29.9)	105 (30.2)	35 (10.0)	0.384
	Detergent	0 (0)	3 (0.8)	0 (0)	
	Antiseptic solution	45 (12.9)	32 (9.2)	23 (6.6)	
Q3: Do you prefer oral mouth rinse before commencement of any treatment procedure?	Yes	117 (33.7)	92 (26.5)	41 (11.8)	0.004
	No	32 (9.22)	48 (13.8)	17 (4.8)	
Q4: Do you think isolation is important in infection?	Yes	139 (40.0)	134 (38.6)	57 (16.4)	0.212
	No	10 (0.2)	6 (1.1)	1 (0.2)	
Q5: With which of the following vaccines have you been vaccinated?	Hepatitis B	35 (10.0)	26 (7.4)	2 (0.5)	0.098
	Tetanus	49 (14.1)	56 (16.1)	19 (5.4)	
	Tuberculosis	62 (17.8)	57 (16.4)	36 (10.3)	
	None	3 (0.8)	1 (0.2)	1 (0.2)	
Q6: Which of the following do you use to sterilize the instruments? In dental clinic?	Autoclave	83 (23.9)	113 (32.5)	56 (16.1)	p < 0.001
	Boiling	56 (16.1)	21 (6.0)	2 (0.5)	
	Washing	10 (2.8)	6 (1.7)	0 (0)	
Q7: Minimum time required for sterilization in autoclave	5 min	7 (2.0)	4 (1.1)	0 (0)	0.001
	10 min	51 (14.6)	25 (7.2)	6 (1.7)	
	15 min	91 (26.2)	111 (31.9)	52 (14.9)	
Q8: Temperature for sterilization in autoclave	100°C	13 (3.7)	8 (2.3)	2 (0.5)	0.038
	120°C	66 (19.0)	67 (19.3)	23 (6.6)	
	150°C	70 (20.1)	65 (18.7)	33 (9.5)	
Q9: Which of the following has the highest rate of transmission via saliva?	Hepatitis B	27 (7.7)	41 (11.8)	17 (4.8)	p < 0.001
	AIDS	33 (9.5)	27 (7.7)	4 (1.1)	
	Tuberculosis	60 (17.2)	69 (19.8)	32 (9.2)	
	Do not know	29 (8.3)	3 (0.8)	5 (1.4)	
Q10: What immediate action should be taken in case of direct blood contact with an HIV patient?	Anti-HIV immunoglobulins	37 (10.6)	41 (11.8)	14 (4.0)	0.019
	Anti-HIV drugs	17 (4.8)	20 (5.7)	5 (1.4)	
	Blood tests	58 (16.7)	61 (17.5)	43 (12.3)	
	Do not know	37 (10.6)	18 (5.1)	5 (1.4)	
Q11: Odds of HIV transmission after a single contaminated needlestick injury?	0.1%–0.4%	12 (3.4)	6 (1.7)	8 (2.3)	0.073
	1%–4%	11 (3.1)	20 (5.7)	9 (2.5)	
	10%–40%	38 (10.9)	29 (8.3)	12 (3.4)	
	70%–90%	88 (25.3)	85 (24.4)	29 (8.3)	
Q12: As a clinician, what protective measures do you take to prevent yourself from injury?	Face mask and gloves	5 (1.4)	11 (3.1)	2 (0.5)	0.009
	Eyewear	23 (6.6)	23 (6.6)	10 (2.8)	
	Protective clothing	5 (1.4)	1 (0.2)	1 (1.4)	
	All the above	116 (33.4)	105 (30.2)	45 (12.9)	
Q13: After use of gloves for a patient, what do you do with them?	Dispose them	148 (42.6)	139 (40.0)	58 (16.7)	0.810
	Reuse them after wash	1 (1.4)	1 (1.4)	0 (0)	
	Reuse them after sterilization	0 (0)	0 (0)	0 (0)	
Q14: Ineffective sterilization during clinical practice can transmit the infection from one patient to another?	Yes	125 (36.0)	132 (38.0)	56 (16.1)	0.001
	No	1 (1.4)	3 (0.8)	1 (1.4)	
	Do not know	23 (6.6)	5 (1.4)	1 (1.4)	
Q15: Apart from instrument sterilization, disinfection of dental chair, clinic, dental office is required?	Yes	132 (38.0)	131 (37.7)	55 (15.8)	0.037
	No	4 (1.1)	3 (0.8)	1 (1.4)	
	Do not know	13 (3.7)	6 (1.7)	2 (0.5)	

^*∗*^Pearson's chi-square test. Level of significance: *p* < 0.05.

**Table 4:** Evaluation of the level of knowledge, attitudes, and practices regarding infection control in undergraduate stomatology students.

Group	Knowledge media (DE)	Attitudes media (DE)	Practices media (DE)
Preclinical	2.47 ± 2.26	3.52 ± 1.00	3.38 ± 1.94
Clinical	2.84 ± 2.16	3.61 ± 0.86	3.58 ± 1.87
Internship	2.93 ± 2.07	3.65 ± 0.78	3.82 ± 1.69
Total	2.74 ± 2.16	3.59 ± 0.88	3.59 ± 1.73

## Data Availability

The data used to support the findings of this study are available from the corresponding author upon request.

## References

[1] Khosravanifard B., Rakhshan V., Najafi-Salehi L., Sherafat S. (2014). Tehran dentists’ knowledge and attitudes towards hepatitis B and their willingness to treat simulated hepatitis B positive patients. *Eastern Mediterranean Health Journal*.

[2] Khosravanifard B., Rakhshan V., Sherafat S., Najafi-Salehi L (2015). Risk factors influencing dentists’ hepatitis B-related knowledge and attitudes and their willingness to treat hepatitis B positive patients. *Eastern Mediterranean Health Journal*.

[3] Carvalho V., Oliveira D., Prado F. (2015). Knowledge, risk perception and attitudes of dentistry students with regard to HIV/AIDS. *RGO–Revista Gaúcha de Odontologia*.

[4] Rahman B., Balu S. H., Mohammed A., Eisa F., Ibrahim S. H. (2013). Attitudes and practices of infection control among senior dental students at college of dentistry, university of Sharjah in the United Arab Emirates. *European Journal of Dentistry*.

[5] Younai F., Murphy D., Kotelchuck D. (2001). Occupational exposures to blood in a dental teaching environment: results of a ten-year surveillance study. *Journal of Dental Education*.

[6] Brailo V., Pelivan I., Škaricic J., Vuletic M., Dulcic N., Cerjan G. (2010). Treating patients with HIV and hepatitis B and C infections: Croatian dental students’ knowledge, attitudes, and risk perceptions. *Journal of Dental Education*.

[7] Machado H., Ramos M., Auad S. H., Laura, Paiva Martins S., Pordeus I. (2008). Occupational exposure to potentially infectious biological material in a dental teaching environment. *Journal of Dental Education*.

[8] Cleveland J. L., Barker L. k., Cuny E. J., Panlilio A. L. (2007). Preventing percutaneous injuries among dental health care personnel. *Journal of American Dental Association*.

[9] Pasha L., Farid H., Faisal M. (2016). Dental professionals experience regarding sharp injuries during practice. *Pakistan Oral and Dental Journal*.

[10] Liege H., Fernandes F., Barbosa W. (2017). Needlestick and sharp instruments injuries among Brazilian dentistry students. *Contemporary Clinical Dentistry*.

[11] Machado H., César T., Martins (2007). Management of occupational bloodborne exposure in a dental teaching environment. *Journal of Dental Education*.

[12] Singh A., Purohit M., Bhambal A., Saxena S., Singh A. N., Gupta A. (2010). Centers for disease control and prevention. knowledge, attitudes, and practice regarding infection control measures among dental students in central India. *Journal of Dental Education*.

[13] Askarian M., Assadian O. (2009). Infection Control practices among dental professionals in shiraz dentistry school, Iran. *Archives of Iranian Medicine*.

[14] Muflih M. (2017). Knowledge, attitudes and practice of infection control among students and interns of college of dentistry, Aljouf University. *International Journal of Medical Research Professionals*.

[15] Sadeghi M., Hakimi H. (2009). Iranian dental students’ knowledge of and attitudes towards HIV/AIDS patients. *Journal of Dental Education*.

[16] Abdullah M. (2011). A survey of needle sticks and other sharp injuries among dental undergraduate students. *International Journal of Infection Control*.

[17] Aggarwal A., Panat S. (2012). Knowledge, attitude, and behavior in managing patients with HIV/AIDS among a group of Indian dental students. *Journal of Dental Education*.

[18] Lopes M., Veras S., Araripe T., Miranda A., Medeiros S., Nunes J. (2013). Hepatitis B: knowledge, vaccine situation and seroconversion of dentistry students of a Public University. *Hepatitis Monthly*.

[19] Pal V., Syazana I., Amanina N. (2017). Knowledge and attitude of dental students towards HIV/AIDS patients in Melaka, Malaysia. *Malaysian Journal of Medical Sciences*.

[20] Khosravanifard B., Rakhshan V., Ghasemi M. (2012). Tehran dentists’ self-reported knowledge and attitudes towards HIV/AIDS and observed willingness to treat simulated HIV-positive patients. *Eastern Mediterranean Health Journal*.

[21] Ahmad A., Sann L, Rahman H. (2016). Factors associated with knowledge, attitude and practice related to hepatitis B and C among international students of Universiti Putra Malaysia. *BMC Public Health*.

[22] Al-Shamiri H. M., AlShalawi F. E., AlJumah T. M., AlHarthi M. M., AlAli E. M., AlHarthi H. M. (2018). Knowledge, attitude and practice of hepatitis B virus infection among dental students and interns in Saudi Arabia. *Journal of Clinical and Experimental Dentistry*.

